# Telephone Referral to a Paediatric Emergency Department: Why Do Parents Not Show Up?

**DOI:** 10.3390/children10020179

**Published:** 2023-01-18

**Authors:** Mélanie Freiermuth, Christopher J. Newman, Judit Villoslada

**Affiliations:** 1 Faculty of Biology and Medicine, University of Lausanne, 1011 Lausanne, Switzerland; 2 Paediatric Neurology and Neurorehabilitation Unit, Lausanne University Hospital, 1011 Lausanne, Switzerland; 3 Paediatric Service, Woman-Mother-Child Department, Lausanne University Hospital, 1011 Lausanne, Switzerland

**Keywords:** telephone triage, non-adherence, children, emergency department

## Abstract

Medical call centres can evaluate and refer patients to an emergency department (ED), a physician or provide guidance for self-care. Our aim was (1) to determine parental adherence to an ED orientation after being referred by the nurses of a call centre, (2) to observe how adherence varies according to children’s characteristics and (3) to assess parents’ reasons for non-adherence. This was a prospective cohort study set in the Lausanne agglomeration, Switzerland. From 1 February to 5 March 2022, paediatric calls (<16 years old) with an ED orientation were selected. Life-threatening emergencies were excluded. Parental adherence was then verified in the ED. All parents were contacted by telephone to respond to a questionnaire regarding their call. Parental adherence to the ED orientation was 75%. Adherence decreased significantly with increasing distance between the place the call originated and the ED. The child’s age, sex and health complaints within calls had no effect on adherence. The three major reasons for non-adherence to telephone referral were: improvement in the child’s condition (50.7%), parents’ decision to go elsewhere (18.3%) and an appointment with a paediatrician (15.5%). Our results offer new perspectives to optimise the telephone assessment of paediatric patients and decrease barriers to adherence.

## 1. Introduction

Medical call centres (CCs) are a valuable aid for parents seeking support for their child’s health when their regular paediatrician is not available [1,2,3]. They have become an integral part of the paediatric care network [4,5] and most importantly, telephone triage appears to be an effective method to prevent unnecessary visits to the emergency department [6,7]. They are managed either by doctors [3,8] or by nurses [9,10,11]. Common models of CCs are operational beyond the opening hours of medical practices [3,4,12]. The majority of CCs use algorithms that dictate questions appropriate to the caller’s complaint. Depending on the answers given by the parents, the algorithm proposes a referral to the most appropriate care setting, i.e., an emergency centre, a primary care physician, or provision of guidance for self-care.

In Europe, the organisation of CCs varies strongly from country to country from publicly funded and organized regional centres to in-house hospital or clinic-based CC triage, through privately run CCs most often affiliated to healthcare insurances and/or providers. For example, in France and Spain, telephone triage is a public remit, organized at a regional or departmental level and considered as a medical act [13,14]. In Denmark and the Netherlands, calls centres are integrated to out-of-hours medical services, from which a physician will answer the phone call, and either provide a consultation on the phone, ask the caller to come for a consultation at the out-of-hours centre or refer the caller to an emergency department. In the UK, CCs are regional, managed by nurses and are available 24/7. In Switzerland, CCs can be public or private, i.e., linked to a health insurance to which the callers must be affiliated.

Parents and paediatric users are usually very satisfied with the services offered by call centres as reported in the USA, England and Australia, particularly in terms of listening and understanding their problem, but also in terms of the guidance they have been offered [2,10,15,16].

Other studies on the subject have focused on the clinical validity of CCs, by assessing patient safety with referrals [17,18], adequacy in terms of referral to an emergency centre [4,9], return on investment [10], the epidemiology of calls [1,12] and parental adherence [8,9,11,16,19] with recommendations from the CC.

The Swiss healthcare system is universal and of a high standard. Everyone living in Switzerland must pay contributions to basic health and accident insurance schemes to receive treatment. Healthcare in Switzerland is largely decentralized and organized by the individual cantons, with both public and private sectors. CCs are organized at a regional or cantonal level. A large majority of children in Switzerland are followed up by a primary care physician (most often a paediatrician) and the country has a high density of public hospitals including paediatric departments offering dedicated paediatric emergency care.

Adherence is an active process of communication between the patient and the healthcare provider where the patient cannot be held totally responsible for non-adherence [20]. The World Health Organization defines it as “the extent to which a person’s behaviour, taking medication, following a diet, and/or executing lifestyle changes, corresponds with agreed recommendations from a health care provider”. Parental adherence after telephone referral varies widely in the literature and depends on the type of orientation offered. For example, some research shows that parents are more likely to go to the emergency department or to stay at home and follow the care advice given than to take an appointment with the on-call paediatrician the next day or later [5,8,21]. Reasons for non-adherence [5,9,15,22] to telephone referrals are represented as a balance between parental perceptions, quality of provider communication and accessibility of the medical service.

Although CCs are increasingly popular and have a high level of user satisfaction worldwide, their impact on the European paediatric population remains unknown. To our knowledge, no European study has examined reasons for parental non-adherence to an emergency centre orientation after a CC referral. Another unexplored aspect in the international literature is how parental adherence varies according to telephone complaints and patient characteristics. Barriers to adherence should be identified with the aim of improving the patients’ outcomes and well-being on one hand, and the quality of health services on the other hand. Knowledge of the outcome of children referred by the CC and of the reasons for non-adherence are important to evaluate the validity of the CC and could improve the management of calls and the quality of telephone assessments. Therefore, the main objectives of this study were (1) to determine parental adherence to a regional paediatric emergency department (ED) after being referred by a regional CC, (2) to observe how adherence varies according to children’s characteristics and (3) to assess the reasons for parental non-adherence.

## 2. Materials and Methods

### 2.1. Study Design

We performed a descriptive and prospective cohort study, including a telephone questionnaire, from 3 February to 5 March 2022.

### 2.2. Participants

The subjects of the study were all children from one day to fifteen years of age who were referred to the regional paediatric ED by the regional CC between 1 February and 5 March 2022.

We excluded all children aged 16 years and over, as well as children with life-threatening emergencies, children referred to their primary care paediatrician, to another emergency centre or who received counselling. Patients meeting the inclusion criteria with incomplete medical records were also excluded.

### 2.3. Setting

#### 2.3.1. Regional Data

The canton of Vaud is the third most populous in Switzerland after Zurich and Bern. The canton’s permanent resident population of Vaud is made up of 824,000 residents (2/3 Swiss nationals, 1/3 foreigners), with 145,000 children aged 0–15 years. Vaud’s capital is Lausanne, and the Lausanne agglomeration covered in the current study includes 51% of the cantonal population, i.e., 420,000 residents and 74,000 children aged 0–15 years, with a population density of approximately 2600 residents/km^2^ [23].

#### 2.3.2. The Centrale Téléphonique des Médecins de Garde

The Centrale Téléphonique des Médecins de Garde is a CC based in Lausanne (canton of Vaud) which responds to medical or social problems concerning the cantons of Vaud and Neuchâtel, 24 h a day. The mission of the CC is to respond to the population’s needs and to direct them towards the best health solution, i.e., to a doctor on duty or to a general practitioner, the nearest emergency centre or to provide advice. Its primary aim is to evaluate, triage and direct each problem to the most appropriate place of care. The triage and orientation of calls are carried out by nurses using triage protocols according to the child’s main complaint. The paediatric assessment consists of two steps: a brief assessment of the child’s condition using the telephone in-house adapted Paediatric Assessment Triangle (PAT) tool [24], followed by the exploration of the severity criteria of the main complaint using pre-established in-house protocols containing targeted and structured questions. The nurse concentrates on the child’s appearance, circulatory status and respiratory work by questioning the parents in the presence of their child. When one of the components is pathological, the child is directly referred to a paediatric ED according to their geographical location, whatever the time of the day and week. The complaints guidelines list the associated symptoms by severity and suggest a referral. They provide freedom to the clinical sense of the nurses taking the call and they can change the referral at any time based on information given by the parents.

Nurses must have prior professional experience in adult or paediatric acute care. They follow specific training for 3 months in triage, medical regulation, and telephone call orientation. Thereafter, courses and further training, as well as annual evaluations and coaching by means of call re-listening, are carried out with the referring doctors.

The CC handled approximately 180,000 calls for the year 2021, of which 67,737 (37.5%) concerned children.

#### 2.3.3. The Hôpital de l’Enfance (HEL) Emergency Department

The HEL is Lausanne University Hospital’s centre of reference in paediatrics for the central area of canton Vaud (Lausanne region). Its ED handled more than 29,000 emergencies for the year 2021 according to annual internal statistics. It takes care of children from birth to 18 years of age, mostly from the Lausanne region. Internal 2018 statistics show that 62% of ED patients are Swiss nationals, 27% are European and 11% extra-European.

### 2.4. Outcomes

Our main outcome was parental adherence to the emergency department orientation, i.e., the number of children who arrived at the ED after being referred by the CC. We also documented the number of patients who arrived at the ED within the recommended time (<2 h).

### 2.5. Variables

The first step was to obtain the list of children whose parents had called on the CC thanks to the SAGASQL software version 5 (Everbridge Inc., Boston, MA, and Los Angeles, CA, USA), a platform that records all the forms filled out by the nurses receiving a call. We collected demographic data (postcode of call origin, date and time of the call, gender, age) and the main call complaint for each participant. Travel distance between the ED and the call location was computed based on the postcode (average driving distance from each postcode). Distance from the ED to the call location was dichotomized into below and equal to or above 10 km (corresponding to the area of highest urban density in the Lausanne agglomeration).

Age was categorised according to the National Institute of Child Health and Human Development age stages [25]: “infancy” birth to 12 months; “toddler” 13 to 24 months; “early childhood” 25 months to 5 years, “middle childhood” 6 to 12 years, “adolescent” 12 to 15 years. Complaints were categorised according to the anatomical classification of B. Schmitt in their Paediatric Telephone Protocols [26]. Anatomic categories were defined as: chest or breathing symptoms, abdomen symptoms, arm and leg symptoms, fever symptoms, skin symptoms, ENT symptoms, crying, genital or urinary symptoms and miscellaneous (eye symptoms, head and brain symptoms, issues with medication, endocrinology, psychiatry, poisoning). If several complaints were described or some data was missing, we proceeded with a replay of the calls to identify the child’s main problem and complete the information.

The second step was to consult the medical file of the children who had been referred to the ED in order to determine whether callers had complied with the orientation proposed by the CC. In addition, the following elements were collected: the date and time of arrival at the emergency service. Parental adherence was defined as arrival within 24 h of the call.

Finally, the parents were all contacted by telephone within 72 h of the call to respond to a structured questionnaire regarding their call, after having given their oral consent. The questionnaire was developed by MF and JV based on the study objectives and their data and experience from the pilot study [27] and was submitted to both adult and paediatric ED senior specialists to ensure face validity before implementation. The questionnaire consisted of 4 multiple choice questions to all participants, and additionally one open question and one multiple choice question to participants who had not adhered to the ED orientation (Table 1).

Responses to the open question on reasons for non-adherence were analysed inductively by MF and JV and categorised in five themes: “child’s condition improved”, “decision to go elsewhere”, “appointment with primary care physician”, “waiting time in emergency department” and “other” (e.g., problems with taking medication resolved in the meantime, relatives working in the care sector doing follow-up at home).

### 2.6. Statistical Analyses

We used an online statistical calculator [28] to estimate adherence in our study population with a precision of 5% and a confidence of 95%; a sample size of at least 281 participants was required, based on data from the same CC and ED, with a 76% adherence measured in a pilot study [27]. This pilot project was developed and conducted by MF and JV as part of a student master thesis and explored parental adherence to an emergency department orientation after telephonic referral and involved a sample of 451 participants over a one-month period (November 2019).

Descriptive statistics were carried out for all call characteristics as well as those of the telephone questionnaires, using SPSS Statistics Version 27.0.1 software (IBM Corporation, Armonk, NY, USA). Categorical data were described in counts and proportions and continuous data (age, distance to ED) were assessed for normality of distribution using the Kolmogorov-Smirnov test. Given the non-normal distribution of both continuous factors, they were described by means, medians and quartiles.

A Mann–Whitney U test was used to explore the association between travel distance from the call location to the ED and adherence to orientation.

Chi^2^ analyses were used to explore bivariate associations between parental adherence and personal characteristics (dichotomized distance, gender, age, main complaint) as well as parents’ answers to the questionnaire (questions 1 to 4) and for significant associations crude odds ratios (OR) with their 95% confidence intervals (CI) were computed. For multivariate analysis, these parameters were then entered in a single step as independent variables into an unconditional binary logistic regression. Dummy variables were created for categorical parameters. *p*-values below 0.05 were considered significant.

## 3. Results

### 3.1. Population, Inclusions and Exclusions

**Figure 1:**
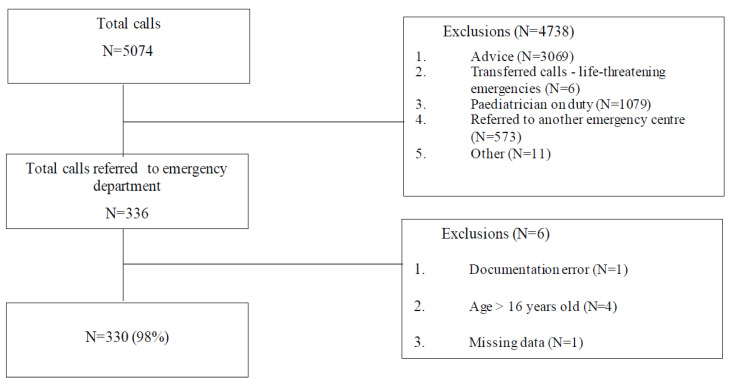
Flowchart of selection and exclusion of calls.

A total of 5074 calls concerning children were recorded by the centre during the study period in canton Vaud. Of these, 3069 (60.5%) received advice, 1079 (21.3%) were booked for an appointment with an on-duty paediatrician, 909 (17.9%) were referred to an emergency centre in the canton, 6 (0.1%) were transferred to the 911 call centre and 11 (0.2%) were calls for training. Three hundred and thirty-six children (6.6%) were referred to the ED by the CC, of whom 330 (6.5%) were included (Figure 1). Six patients were excluded: one for a documentation error in the software, four were older than sixteen years and one had missing data (name, date of birth).

### 3.2. Parental Adherence

Of the 330 patients included, 247 presented at the emergency department. Parental adherence to the ED referral after calling the CC was 75%. Of the 247 patients, 219 (89%) arrived at the emergency department within 2 h of the call.

### 3.3. Characteristics of Callers and Adherence

Forty-six percent (151/330) of the children requiring referral from the CC were female. Parental adherence by gender was 74% for boys and 76% for girls.

The median age of the children for whom parents called was 2 years (mean = 4.5 years, P25 = 0 years, P75 = 6 years).

The three main complaints in telephone calls were chest or breathing symptoms (97/330, 29.4%), abdomen symptoms (70/330, 21.2%) and arm and leg symptoms (44/330, 13.3%).

Table 2 shows parental adherence according to complaint category.

Parental adherence and main complaints according to their child’s age category are described in Table 3.

There were no significant differences between adherent and non-adherent parents in univariate or multivariate analyses for child gender, age category or main complaints.

**Figure 2:**
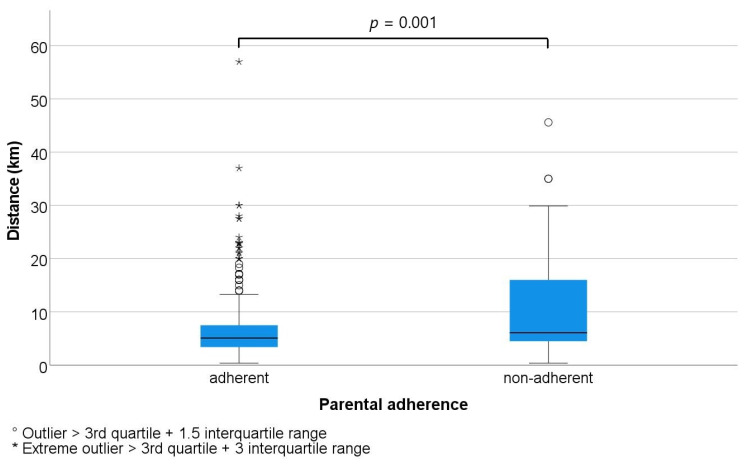
Parental adherence according to distance from their location to the paediatric emergency department.

Median travel distance to the ED was 5.1 km (mean = 7.3 km, P25 = 3.4 km, P75 = 7.5 km) for families who adhered to the orientation, compared to a median 6.1 km (mean = 11.0 km, P25 = 4.5 km, P75 = 16.0 km) for parents who did not follow the referral, with a highly significant difference in distribution (*p* = 0.001). Figure 2 shows the adherence according to the distance between the ED and the place of call.

Families that were within 10 km travel distance of the ED were significantly more likely to adhere to the ED orientation (201/252, 80%) than families who were 10 km or beyond (46/78, 59%) with a crude odd ratio of 2.7 (95%CI: 1.6–4.7; *p* < 0.001)) in the bivariate analysis and a corrected odd ratio of 3.5 (95%CI: 1.8–6.6; *p* < 0.001) in the multivariate analysis.

### 3.4. Parental Perceptions and Reasons for Non-Adherence

Of the 330 parents subsequently contacted by telephone, 283 took part in the telephone questionnaire (participation rate 86%).

A majority (73.9%) of parents called the CC because they didn’t know what to do and needed some orientation, 17.3% called for general advice and 8.8% wanted an appointment with the paediatrician on duty.

Communication was rated as excellent by 37%, very good by 42%, good by 19% and poor by 2%.

Ninety-eight percent (278/283) of the callers had a primary care paediatrician for their child.

At the time of the call, a summary of the child’s situation was made by the nurse for 59% of parents (166/283). A total of 12% of the parents (33/283) had no recollection of a summary by the nurse and 30% said they had not received a summary (84/283).

None of these factors (call expectation, communication quality, primary care paediatrician, complaint reformulation by CC nurse) was significantly associated with adherence in bivariate or multivariate analyses.

**Figure 3:**
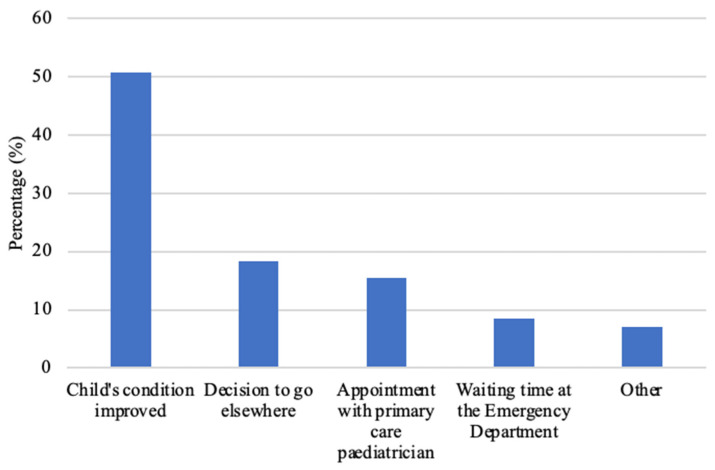
Parental reasons for non-adherence, expressed in percentage (%, *n* = 71).

Figure 3 shows the reasons for non-adherence given by parents who did not comply with the CC advice.

## 4. Discussion

Parental adherence to CC recommendations with referral to the ED was 75%. This result is consistent with American studies published to date that report adherence rates between 63% and 86% after telephone referral by nurses [5,9,11,16,19]. It is difficult to objectively determine whether adherence is satisfactory, as there are to date no benchmarks on quality criteria to determine the effectiveness or usefulness of a CC. Clinical validity of a CC can be assessed in two ways: internal validity, which assesses patient safety by comparing the degree of urgency described in the CC with the degree observed once the patient arrives at the ED, and external validity, which compares the same data in different care settings [17,18,29]. However, currently no consensus has been reached on the precise criteria to determine the urgency of each situation during triage [29]. The effectiveness of a paediatric CC may also be defined by its mission and objectives. The mission of our CC was to refer children at risk of a less favourable health outcome if they were not seen in an ED within the 2 h following the call. On this basis, the telephone triage system was expected to over-triage, i.e., not to underestimate the severity of the child’s condition, in order to avoid potentially dangerous situations for the patients. Thus, when in doubt about a telephone situation and without visual access to the child, a nurse will favour that the child is assessed in the ED. In our study, when parents were asked why they did not go to the ED, half of them reported that since their child’s condition improved after the call, they no longer considered it necessary to attend. This has been previously described [5,9,22]. Further studies measuring the correlation between adherence to and the clinical validity of paediatric telephone triage could prove useful to determine the optimal level of adherence to a CC, providing a benchmark for paediatric CCs. Internal validity is analysed in some studies by assessing agreement between the triage system urgency and a reference urgency by using kappa statistics [30], and weighing in individual adherence as measured in the current study could further strengthen such analyses. In addition, as a potential straightforward benchmark of CCs, adherence data could help to determine the value of paediatric phone triage systems in different settings, thus improving knowledge of their external validity.

Of all the parents who went to the ED, a majority respected the recommended 2 h time limit to get to the hospital, which demonstrates good overall communication and a good understanding of the information transmitted by the centre to parents. It also reflects the trust that most parents place in the information provided by the CC and in the care system in general. This is also underlined by the satisfaction rate in the telephone survey, where 98% of parents rated the communication as good-to-excellent, noting also that they felt seriously considered in their request for help, with results comparable to previous studies [10,16]. In this context, the CC fulfils its mission of providing guidance and responding to a need of the population.

Another reason for non-adherence given by parents was that the proposed ED was not suitable, either because of previous negative experiences with the centre or because parents were at the same distance from another emergency centre, and so preferred to go elsewhere, which was also found in the study by Kempe and al. [9]. Certain parents were seen at a later stage by a primary care paediatrician, while others stayed at home due to the waiting time at the ED. Why could a situation that is considered urgent over the telephone not be acknowledged as such by certain parents, if judging by their subsequent behaviour? Lack of clarity of the nursing advice has been demonstrated to have an influence on the parental decision to visit the emergency department; parental perceptions of the illness, their motivation and their trust in the call service also have an influence on adherence [5]. Purc-Stephenson et al. [5] emphasised the role of communication as a factor in non-adherence and described the importance of explaining to parents the reasons for an emergency room visit. Mayo et al. [31] note that in the telephone triage steps, assessing parents’ understanding of the proposed referral is the least frequently performed step. This was confirmed by our telephone survey, in which more than a third of parents said they had not had a summary of their child’s situation or could not remember it, which could explain the lack of understanding of some of the CC’s instructions. Even if our study showed no association between reformulation of the complaint by the nurse and adherence to ED orientation, we believe this is an important step to consider, since the implementation of a systematic process to verify the understanding of the information received is likely to support parental adherence. This can easily be extended to other territories and throughout sociodemographic categories, and implementation can be consolidated by generating institutional learning, ingraining it into local regulatory systems and protocols, thus strengthening telephone screening and helping conversation. It does, however, require that professionals learn how to check that parents have understood.

The distance between place of call and place of care played a significant role in parental adherence (*p*-value = 0.001). Indeed, the further away parents were from the ED recommended by the CC, the less likely they were to go there. Accessibility of an ED has been demonstrated to be impacted by the lack of transportation [5]. Our study demonstrated that distance to the ED per se can be considered as a barrier to access to care: the greater the distance, the lower the adherence. This finding has a bearing on the current trend towards centralising care facilities. Although the aim is to improve the quality of care by increasing critical mass, distance to care seems to be a hindrance for some parents living in decentralised areas [32]. For parents who cannot easily travel to the ED, it may be appropriate to introduce video-call triage or telemedical consultations to improve access to care. Recent studies have shown a real benefit both in terms of adherence to recommendations [33] and in terms of patient safety [34]. Haimi et al. [34] reported high rates of appropriate diagnosis and reasonable treatment decisions, with 82% sensitivity and 96% specificity.

In our study, the age and gender of the child had no influence on parental adherence, a result comparable to the literature [8,35], except Scarfone and al. [11], who observed a significantly higher adherence for younger children. The type of complaint had no significant impact on the parental decision to consult the ED. The emergency was taken just as seriously by parents, whether a trauma, abdominal pain or respiratory distress. However, it is important to bear in mind that sample sizes varied for different categories of complaints and could have limited our ability to detect statistically significant differences for the smaller groups. Parents tended to be more likely to visit the ED for urogenital complaints, and less for unspecific complaints such as crying. Specific complaint patterns emerged according to age, especially for the infant category. These patterns that differ from adult complaints (according to Thierrin et al. [36] the main complaints of adults at the same CC were digestive, altered general condition and ENT issues) can raise age-specific awareness and improve the training of CC respondents. They also question whether on-site attendance of professionals with clinical experience in acute paediatrics would benefit a CC. Regardless of age group, respiratory complaints were the most frequently mentioned by parents, similar to previous studies [11,37].

Our study presented several limitations, among which is the reliability of the data collected in the software of the CC. The notes concerning parental complaints entered by the nurses were not standardised in the software, making the extraction of data difficult. Furthermore, as many CCs do not use pre-established in-house protocols but rather decision-triage algorithms, our data may not be generalizable to all CCs. Our brief telephone questionnaire was purposively developed in-house to respond to the study objectives and superficially assessed for face validity by two professionals independent from the study conduct; however, it was not formally tested for psychometrical robustness (i.e. construct, content and criterion validities, reliability). It should also be mentioned that this study focuses on the Lausanne agglomeration population for reasons of hospital territorial organisation. Despite the demographic and multicultural composition of this region being similar to neighbouring territories, our results may not be generalizable to other regions. We did not account for socio-demographic factors such as family income, parent education, literacy or nationality for lack of robust access to this information and having elected to exclude this from the telephone questionnaire due to feasibility and acceptability issues, and it is possible that any one of these factors could have borne on adherence and beforehand on parents’ access to the CC. For example, one of the important dimensions of phone advice related to issues of trust between the nurse and parents may be influenced by cultural and ethnic issues [38]. Finally, as previously mentioned, our sample size may have limited our ability to infer certain associations, especially for less frequent complaint groups.

## 5. Conclusions

This study is the first in Europe to examine why parents decide not to go to the ED after a telephone referral from a CC and to specifically examine in combination child, parent and call characteristics that could determine adherence. Parental adherence to CC recommendations varied according to the distance from the ED, but not according to the child’s age, sex or complaint, nor to parental call expectations, to quality of the communication (which was very largely judged good-to-excellent), to the reformulation of the complaint by the CC nurse, or whether the child had a primary care paediatrician or not. Children presented different patterns of complaints according to their age category. In a context where medical CCs are constantly developing, our study offers new perspectives to optimise the telephone assessment of paediatric patients and to improve call structure. Moreover, professionals with clinical experience in paediatrics could provide a real advantage in terms of assessment and telephone screening but also in improving the quality of phone communication.

## Figures and Tables

**Table 1:** Telephone questionnaire.

Question	Answers
What were your expectations when you called the medical call centre?	Get an orientation Get advice Get an appointment with an on-duty paediatrician
2. How did you find communication with the nurse?	Excellent Very good Good Poor
3. Did the nurse check your understanding of the situation and the need to go to the emergency room by having you repeat important points or by summarising the situation?	Yes No Don’t remember
4. Do you have a treating paediatrician?	Yes No
If non adherence to emergency department orientation	
5. We have not recorded your attendance in the emergency department, why did you not go there?	Free response
6. Did you make a telephone contact/get an appointment with your treating paediatrician in the meantime?	Yes No

**Table 2:** Adherence rates overall and by category of complaint.

	M/N	Adherence (%)
TOTAL	247/330	75
Chest or breathing symptoms	75/97	77
Abdomen symptoms	55/70	79
Arm and leg symptoms	36/44	82
Fever symptoms	25/34	73
Skin symptoms	18/28	64
ENT symptoms	14/20	70
Crying	7/12	58
Genital or urinary symptoms	9/10	90
Miscellaneous	8/15	53

Overall adherence to the paediatric emergency department and according to the category of complaint, expressed in counts (M: ED visits; N: calls) and proportions (%).

**Table 3:** Main telephone complaints and adherence rates to the paediatric emergency department referral by age group.

Groups	Age	Main Complaints	Adherence (%)
Infant (*n* = 108)	0–12 mo	Chest or breathing symptoms (n = 44, 41%)	82
		Fever symptoms (n = 20, 19%)	
		Abdomen symptoms (n = 15, 14%)	
Toddler (*n* = 44)	13–24 mo	Abdomen symptoms (n = 10, 23%)	68
		Chest or breathing symptoms (n = 9, 21%)	
		Arm and leg symptoms (n = 7, 16%)	
Early childhood (*n* = 93)	25 mo–5 y	Chest or breathing symptoms (n = 28, 30%)	72
		Abdomen symptoms (n = 24, 26%)	
		Arm and leg symptoms (n = 14, 15%)	
Middle childhood (*n* = 60)	6–12 y	Abdomen symptoms (n = 15, 25%)	72
		Chest or breathing symptoms (n = 12, 20%)	
		Arm and leg symptoms (n = 12, 20%)	
Adolescent (*n* = 25)	12–15 y	Abdomen symptoms (n = 6, 24%)	72
		Arm and leg symptoms (n = 5, 20%)	
		Chest or breathing symptoms (n = 4, 16%)	

## Data Availability

The data presented in this study are available upon request from the corresponding author.
